# Sex differences in alcohol inhibits bone formation and promotes bone resorption in young male and female rats by altering intestinal flora, metabolites, and bone microenvironment

**DOI:** 10.1371/journal.pone.0323222

**Published:** 2025-05-08

**Authors:** Ming Cheng, Hua Lu, Yangling Wu, Long Jia, Tao Xiang, L.i Deng, Guanlan Zhao, Junwei Feng

**Affiliations:** 1 School of Sports Medicine and Health, Chengdu Sport University, Sichuan, China; 2 Department of Rehabilitation, Jinniu District People’s Hospital of Chengdu, Sichuan, China; 3 Operating room, Sichuan Academy of Medical Sciences& Sichuan Provincial People’s Hospital, Sichuan, China; 4 Department of Orthopaedics, Sichuan Academy of Medical Sciences& Sichuan Provincial People’s Hospital, Sichuan, China; University of Life Sciences in Lublin, POLAND

## Abstract

**Background:**

Long-term alcohol intake has toxic effects on osteoblasts and osteoclasts, resulting in decreased bone density, which directly disrupts the composition of the gut microbiota and affects bone metabolism and immune activity. The effects of alcohol on the bones may be closely related to sex. This study investigated the effects of long-term alcohol consumption on bone status in different sexes by examining the gut microbiota, bone metabolism, and immune activity.

**Methods:**

Young male and female rats were administered a Bio-Serv liquid diet containing 5% alcohol. The effects of alcohol metabolism capacity, bone morphology, bone formation, bone resorption, bone marrow immune activity, gut microbiota, and metabolite differences were analyzed in male and female rats using hematoxylin and eosin staining, micro-computed tomography, enzyme-linked immunosorbent assay, western blotting, 16S rRNA sequencing, and untargeted metabolomics.

**Results:**

Chronic alcohol consumption resulted in excessive osteoclast activation and decreased bone mineral density. Furthermore, alcohol reduced bone metabolism and formation while increasing bone resorption. Bone loss was significantly more severe in female rats than in male rats, indicating that the effects of alcohol on rat bones are related to sex. Chronic alcohol consumption also led to polarization of bone marrow immunoreactivity toward the M1 phenotype. In addition, chronic alcohol consumption affected the composition of gut microbiota, reduced the richness and diversity of intestinal microbiota, and decreased the ratio of *Firmicutes*/*Bacteroidetes*. Long-term alcohol consumption also affected fecal metabolites, and 754 differentially expressed metabolites were identified.

**Conclusions:**

Chronic alcohol consumption increased bone resorption, inhibited bone formation, and affected bone marrow immunoreactivity in young male and female rats. Alcohol can also affect gut microbiota composition and fecal metabolism. Female rats were more susceptible to alcohol, possibly because young female rats have a lower alcohol metabolism, immunomodulatory capacity, and gut microbiota diversity than young male rats.

## Introduction

Alcohol abuse is a global public health concern. Chronic alcohol consumption can cause various degrees of damage to various organs such as the brain, heart, liver, muscles, and bones, and can even induce alcohol-related diseases [1]. Notably, chronic daily alcohol consumption (intake of ≥ 3 units per day, with each standard drink equal to 14 g or 0.6 ounces of pure alcohol) is a risk factor for osteoporosis [2–4]. Proper alcohol consumption (approximately 11–77 g/week) may promote bone growth, but excessive alcohol consumption may eventually lead to bone loss or even osteoporosis (OP) [5]. Studies have shown that chronic alcohol intake exerts toxic effects on osteoblasts and osteoclasts, resulting in decreased bone density [6]. Bone loss due to alcohol-induced injury can ultimately lead to osteopenia [1]. Furthermore, chronic alcohol intake and alcoholism are harmful to bone tissue and increase the risk of osteoporosis and osteonecrosis [7,8]. Our previous study found that older rats with chronic alcohol intake were more susceptible to osteoporosis than young rats. However, this does not mean that young rats are entirely resistant to alcohol [9]. Alcohol has been found to affect bone mineral density differently in men and women [10]; therefore, we hypothesized that there may be sex differences in the effects of alcohol on bone. In fact, although studies have shown that adolescent alcohol consumption has declined substantially in many high-income countries over the past two decades [11], it cannot be ignored that adolescent alcohol consumption is still widely recognized as a risk factor for harm to health and society. Alcohol consumption may affect bone development as adolescents mature. Not only age-related primary OP, studies have reported that excessive alcohol consumption is one of the risk factors for secondary OP [12,13]. Additionally, sex issues can be a concern in cases of severe alcohol abuse [14]. Osteoporosis often increases exponentially with age and is predominant in women, but the incidence in men cannot be ignored [15,16]. Physiological and anatomical differences cause women to absorb greater amounts of alcohol and metabolize it more slowly than men [17]. These differences may lead to greater adverse effects and health risks in women who drink alcohol.

Excessive chronic ethanol consumption has been shown to directly damage the composition of the gut microbiota [18]. Recent studies have shown that gut microbial dysbiosis may cause bacterial metabolites to secrete pro-inflammatory factors, thereby affecting bone health [19], and impairment of the intestinal barrier caused by gut microbiota disturbance promotes the pathogenesis of osteoporosis [20]. Dysregulation of the gut microbiota affects bone healing and remodeling, and contributes to osteoporosis and osteopenia [21]. Furthermore, studies have reported sex differences in the gut microbiota [22], although it is unclear whether these differences contribute to differences in alcoholic osteoporosis. Moreover, the gut microbiota affects bone metabolism and immune activity [23,24]. We hypothesized that chronic alcohol consumption affects bone formation and resorption in young rats, possibly by affecting gut microbial composition, metabolism, and immune activity, and that this effect may be closely related to sex.

In order to verify this hypothesis, in this study, male and female young rats were selected as a comparison, and alcohol metabolism capacity, bone formation, bone resorption, bone marrow immune activity, changes in gut microbial diversity, and differences in metabolites of intestinal flora between male and female rats were compared after long-term alcohol consumption, to investigate the effects of alcohol on bone formation in young rats of different sexes. This study establishes a research foundation for preventing the effects of alcohol on bone development in adolescents.

## Materials and methods

### Animals

Twelve 8-week-old Sprague-Dawley (SD) male rats and twelve 8-week-old SD female rats (body weight 200 ± 20 g) purchased from Chengdu Dashuo Biotechnology Co., Ltd (Sichuan, China) were adapted in standard pathogen-free environment for one week. All animals were exposed to 12 h of light and dark. The rats were housed in autoclaved microisolator cages and provided autoclaved food and sterile water ad libitum. Animal experiments were approved by the Chengdu Jinniu District People’s Hospital (ethics number: QYYLL-2022–016) and performed in accordance with the ARRIVE guidelines.

### Experimental design

The alcohol-treated rat model was employed as previously described with some modiﬁcations [9,25]. SD rats were randomly divided into four groups (*n* = 6): (**a**) male normal rats (MN), (**b**) male alcohol-treated rats (MA), (**c**) female normal rats (FN), and (**d**) female alcohol-treated rats (FA). The MA and FA groups were gavaged with the Bio-Serv Liquid Rat Diet LD82 containing 5% alcohol (Bio-Serv, Frenchtown, NJ, USA) for 12 weeks. The alcohol content was 3% (21% of total energy) on days 1 and 2, 4% (28% of total energy) on days 3 and 4, and 5% (36% of total energy) on and after day 5 and was maintained for 12 weeks. The MN and FN groups were fed an isocaloric liquid diet containing dextrin-maltose (food free of phytoestrogens). Rat feces were collected for sequencing of intestinal flora and fecal metabolites. Rats were housed in metabolic cages to collect fresh feces. The fecal samples were collected using sterile forceps, placed in sterile tubes, and immediately stored at −80°C. Before extracting whole blood, the rats were anesthetized using isoflurane at a concentration of 2–3%. The collected blood was allowed to stand for 60 min at room temperature and centrifuged at 3000 rpm for 10 min at 4°C after clotting to obtain serum. Subsequently, the rats were sacrificed by cervical dislocation, and distal femur and bone marrow samples were collected for further analysis. Part of the distal femur tissue was stored in 4% paraformaldehyde (PFA) for tartrate-resistant acid phosphatase (TRAP) staining and hematoxylin and eosin (H&E) staining. Bone marrow samples were obtained by destroying femurs and stored at −80°C until western blotting and flow cytometry analyses.

### TRAP staining

Mature osteoclasts were stained for TRAP. The distal femurs tissues (sections) were fixed with 4% PFA fixation for 48 h and fully decalcified using 15% ethylenediaminetetracetic acid (EDTA) for 3 weeks, then 5 μm thick paraffin sections of femoral tissues were prepared. TRAP staining of osteoclasts in bone tissues was performed using a TRAP Staining Kit (P0332; Beyotime Institute of Biotechnology, China) according to the manufacturer’s instructions. Incubate at 37°C for 1 h in the dark, counterstain with hematoxylin, reverse blue with tap water, and observe the staining effect under a light microscope. The osteoclasts or cytoplasm showed a wine-red color with a light-blue background. Cells with more than three nuclei and TRAP cytoplasmic staining (red) were considered osteoclasts. Bone trabeculae within 3 mm of the growth plate were divided into three regions (1 mm each), and the number of TRAP+ cells in each region of interest (ROI) was counted using the Cell Counter ImageJ plugin (NIH, Bethesda, MD, USA).

### H&E staining

H&E staining was performed to observe bone tissue morphology. Femoral tissues were cut into 3–5 μm slices. Subsequently, deparaffinized and dehydrated sections were stained with hematoxylin and eosin (Service Bio, Wuhan, China). Finally, changes (characterized by thinning of the bone trabeculae, enlargement of the bone marrow cavity, and reduction in hematopoietic cells) in the femoral tissue structure in each group were observed using a microscope (Zeiss AxioVision, Germany). Femoral tissue sections were viewed using light microscopy at low magnification, and 200 × and 400 × images were taken from the selected areas to observe specific lesions.

### Micro computed tomography (Micro-CT) scan

Micro-computed tomography (micro-CT) was used to examine rat femurs. Each sample was scanned using Micro-CT scanner software (Bruker SkyScan1176, Germany). The scanning conditions: scanning voltage was set to 50 kV, scanning current was 500 μA, the scanning resolution was 12 μm, the images were taken at a resolution of 1024 × 1024, and the exposure time was 1600 ms, with 1 mm aluminum filter and 0.3° rotation step. The ROI of the distal femur was 3.0 mm wide, starting 1.0 mm from the proximal end of the distal femoral growth plate. Three-dimensional images were reconstructed using the N-Recon software. For the 3D analysis, the trabecular bones were analyzed using CT-AN software (Bruker Micro-CT) to obtain the following parameters: structure model index (SMI), bone mineral density (BMD), bone volume to total volume ratio (BV/TV), and connectivity density (Conn. Dn), and trabecular separation (Tb. Sp).

### ELISA

Rat serum was collected for enzyme-linked immunosorbent assays (ELISA) testing. The serum was centrifuged at 3000 × *g* for 10 min, and the supernatant was collected for detection. The levels of alcohol dehydrogenase (ADH), aldehyde dehydrogenase (ALDH), bone alkaline phosphatase (BALP), tartrate-resistant acid phosphatase form 5b (TRAP-5b), osteocalcin (OCN), calcitonin (CT), osteoprotegerin (OPG), and insulin-like growth factor 1 (IGF-1) were examined using ELISA kits, according to the manufacturer’s instructions. Rat ADH ELISA Kit (ZC-37527), Rat ALDH ELISA Kit (ZC-37529), Rat BALP ELISA Kit (ZC-36662), Rat TRACP-5b ELISA Kit (ZC-36867), Rat OCN ELISA Kit (ZC-36660), Rat CT ELISA Kit (ZC-36806), Rat OPG ELISA Kit (ZC-36651), and Rat IGF-1 ELISA Kit (ZC-37513) were provided by Shanghai ZCIBIO Technology Co., Ltd. (Shanghai, China). OD values were measured at a wavelength of 450 nm, according to the manufacturer’s instructions. The detection limits and CV of the intra- and inter-assays of the ELISA kits used are listed in S1 Table.

### Spectrophotometry

Calcium (Ca) and phosphorous (P) levels in the rat serum were detected using spectrophotometry according to the manufacturer’s instructions. The Serum Calcium Assay Kit (ZC-S0722) and Total Phosphorus Assay Kit (ZC-S0888) were purchased from Shanghai ZCIBIO Technology Co., Ltd. (Shanghai, China). The absorbance of P was measured at 660 nm, and Ca levels were detected using a colorimetric method.

### Flow cytometry

The numbers of CD80+ (M1 polarized macrophages) and CD206+ (M2 polarized macrophages) cells in the bone marrow were analyzed by flow cytometry. The rat femoral bone cavity was repeatedly washed with PBS, the cell suspension was collected and centrifuged at 300 × *g* for 5 min, the supernatant was discarded, and the red blood cell lysate (G2015-500ML, Servicebio, USA) was added at three times the volume and split at room temperature for 5 min. The cells were washed twice with PBS and the cell precipitate was collected by centrifugation for later use. The cells were then incubated with PE anti-rat CD80 antibody (No. F3108002, Multi Sciences, China; and FITC anti-mannose receptor antibody, No. ab8918, Abcam, China) in the dark for 30 min. The suspensions were washed with PBS, and then stained with 5 μL V-PE or 5 μL V-FITC at room temperature for 15 min in the dark. After staining, data were obtained by flow cytometry using a BD FACSVerse flow cytometer (BD Biosciences, San Jose, CA, USA), and CD80 + and CD206 + cells were analyzed using the CytExpert software (Beckman Coulter Life Sciences, Danvers, MA, USA).

### Western blot analysis

The protein expression levels of inducible nitric oxide synthase (iNOS, M1 polarization marker), cyclooxygenase-2 (COX-2, M1 polarization marker), CD163 (M2 polarization marker), and Arginase-1 (Arg-1, M2 polarization marker) in the bone marrow were measured by western blotting. Total protein was extracted using radioimmunoprecipitation assay (RIPA) lysis buffer (1:10, Servicebio, China), and protein concentrations were determined using a BCA protein quantification kit (Beyotime Institute of Biotechnology, China). Sodium dodecyl sulfate-polyacrylamide gel electrophoresis (SDS-PAGE) was performed to separate proteins using 10% (v/v) polyacrylamide gels at 100 V for 15 min. When the bromophenol blue dye reached the separation gel, the voltage was adjusted to 180 V and the electrophoresis continued for 30–40 min. The electrophoresis was terminated when it reaches the bottom of the separation gel. The membrane was then transferred to a polyvinylidene fluoride (PVDF) membrane (200 mA, 2 h), 5% skim milk diluted in TBST buffer was added, and the mixture was gently shaken for 2 h. The PVDF membranes were placed into primary antibodies (iNOS, ab3523, 1:1000, Abcam; COX-2, ab179800, 1:2000, Abcam; CD163, ab182422, 1:1000, Abcam; Arg-1, ab133543, 1:2000, Abcam; β-actin, AC026, 1:50000, Abclonal) and incubated overnight at 4°C. The PVDF membranes were then mixed with goat anti-rabbit IgG (H + L) (AS014, 1:5000, Abclonal) and incubated at room temperature for 2 h. Primary and secondary antibodies were diluted in 3% bovine serum albumine (BSA)/ PBS. The films were developed with ECL chemiluminescence solution (Beyotime, Shanghai, China). Finally, the proteins were observed on a Tanon-5200 Chemiluminescent Imaging System (Shanghai Tanon GIS 5200, China), and the relative protein expression of the target protein to *β*-actin was determined.

### Gut microbiota analysis

Feces from each group of rats were collected and total DNA was extracted using a fecal genome DNA extraction kit (DP328–02, Tiangen, China). DNA was quantified using a Nanodrop spectrophotometer (Thermo Fisher Scientific, Waltham, MA, USA) and DNA quality was detected using 1.2% agarose gel electrophoresis. The following primers were used for PCR amplification of the V3-V4 region of the 16S rRNA gene, forward primer 338F: 5′-ACTCCTACGGGAGGCAGCA-3′, and reverse primer 806R: 5′-GGACTACHVGGGTWTCTAAT-3′. The recovered PCR amplification products were quantified by fluorescence using a Quant-iT PicoGreen dsDNA Assay Kit and the Microplate reader (BioTek, FLx800). Sequencing libraries were prepared using a TruSeq Nano DNA LT Library Prep Kit (Illumina, USA). After establishing the libraries, quality testing was performed using an Agilent High-Sensitivity DNA Kit (Agilent Technologies, USA). The qualified libraries had only a single peak and no junctions. Finally, paired-end sequencing was performed on an Illumina MiSeq platform. Raw sequencing data were obtained using QIIME2 DADA2 (2019.4) software (https://docs.qiime2.org/) for quality filtering, sequence denoising, splicing, chimera removal, and data analysis. PICRUSt2 was used to predict the 16S rRNA gene sequences in multiple functional databases, including KEGG (https://www.kegg.jp/) and Metacyc (https://metacyc.org/). Graphs were generated using the R package ggplot2.

### Fecal metabolomics analysis

The effects of alcohol on fecal metabolism in male and female rats were studied using untargeted metabolomics. Each 100 mg sample of feces samples was added into a 2 mL centrifuge tube with 600 µ L MeOH (stored at −20°C) (Containing 2-Amino-3-(2-chloro-phenyl)-propionic acid (4 ppm) and vortexed for 30 s. Add 100 mg glass bead (Sigma-Aldrich, Shanghai, China), placed in a tissue grinder (Zhejiang Meibi Experiment Equipment Co., Ltd, Zhejiang, China) for 90 s at 60 Hz. Centrifuged for 10 min at 12,000 rpm and 4°C, filtered the supernatant by 0.22 μm of polytetrafluoroethylene (PTFE) membrane (Jinteng, Tianjin, China) and transfered into the detection bottle for LC-MS detection. LC analysis was performed using an ACQUITY UPLC System (Waters, Milford, MA, USA). Chromatography was carried out with an ACQUITY UPLC HSS T3 (150 × 2.1 mm, 1.8 µm) (Waters, Milford, MA, USA). The column maintained at 40°C. The flow rate and injection volume were set at 0.25 mL/min and 2 μL, respectively. For LC-ESI (+) - MS analysis, the mobile phase consisted of (C) 0.1% formic acid in acetonitrile (v/v) and (D) 0.1% formic acid in water (v/v). Separation was conducted under the following gradient: 0–1 min, 2% C; 1–9 min, 2–50% C; 9–12 min, 50–98% C; 12 ~ 13.5 min, 98% C; 13.5 ~ 14 min, 98–2% C; and 14–20 min, 2% C. For LC-ESI (-) - MS analysis, the analytes were carried out with (A) acetonitrile and (B) ammonium formate (5 mM). Separation was conducted under the following gradient: 0–1 min, 2% A; 1–9 min, 2%–50% A; 9–12 min, 50%–98% A; 12–13.5 min, 98% A; 13.5–14 min, 98%–2% A; 14–17 min, 2% A.

Mass spectrometric detection of metabolites was performed using a Q Exactive (Thermo Fisher Scientific) with an ESI ion source. Simultaneous MS1 and MS/MS (Full MS-ddMS2 mode, data-dependent MS/MS) acquisitions were performed. The parameters were as follows: sheath gas pressure, 30 arb; aux gas flow, 10 arb; spray voltage, 3.50 kV and −2.50 kV for ESI (+) and ESI (-), respectively; capillary temperature, 325°C; MS1 range, *m/z* 81–1000; MS1 resolving power, 70000 FWHM; number of data dependant scans per cycle, 10; MS/MS resolving power, 17500 FWHM; normalized collision energy, 30%; dynamic exclusion time, automatic. To obtain reliable and high-quality metabolomic data, the experiments were validated using quality control (QC) and assurance (QA). Metabolite identification was first confirmed based on exact molecular weight, and then, according to MS/MS fragmentation patterns, the Human Metabolome Database (HMDB) (http://www.hmdb.ca), Massbank (http://www.massbank.jp/), LipidMaps (http://www.lipidmaps.org), mzcloud (https://www.mzcloud.org), and self-built standard databases were used to identify differential metabolites. Metabolites were identified using orthogonal partial least squares discriminant analysis (OPLS-DA). UPLC-MS/MS analyses were performed, and differential metabolites with VIP > 1 and *p* < 0.05 were selected as potential biomarkers. In addition, KEGG analysis was performed to analyze differential metabolite pathways.

### Statistical analysis

Statistical analyses were performed using SPSS software (version 25.0; IBM Corp., Armonk, NY, USA). Data are presented as mean ± standard deviation (SD). Data were analyzed using two-way analysis of variance (ANOVA) to determine the effects of sex and alcohol consumption on specific dependent variables and their interactions. If there was a significant main effect or interaction, the Bonferroni post hoc correction test was used for multiple comparisons. Statistical significance was defined as *p *< 0.05.

## Results

### Effect of alcohol on bone morphology in young female and male rats

With regards to osteoclast number, there were significant main effects of alcohol (F = 24.009, *p* = 0.001, η^2^ = 0.750), but there was no significant sex × alcohol interaction (F = 1.923, *p *= 0.203 > 0.05, η^2^ = 0.194) and no significant sex main effects were found (F = 1.444, *p *= 0.264 > 0.05, η^2^ = 0.153) (S2 Table). TRAP staining showed that the number of osteoclasts in each ROI in female and male chronic alcohol-drinking rats was significantly higher than that in the female and male control groups (*p *< 0.05, *p *< 0.01) (**Fig 1A**, **1B**). This suggests that chronic alcohol consumption leads to bone resorption, which is characterized by osteoclast overactivity. The bone tissue of rats was stained with H&E, and histopathological results showed that the bone trabecular structures of male and female rats were significantly damaged after long-term alcohol consumption; the trabecular bone was partially broken, the bone marrow cavity was enlarged, and a decrease in bone marrow hematopoietic cells was observed in female long-term alcohol consumption rats (**Fig 1C**). To further clarify the effects of long-term alcohol consumption on rat bone tissue, we measured femur trabecular thickness using micro-CT. The two-dimensional (2D) and 3D structure maps of the micro-CT of the femur in each group are shown in **Fig. 1D**. The 2D and 3D structures of the femur of rats in the MN and FN groups were intact, and the 2D and 3D structures of the femur in the MA and FA groups were significantly damaged. The results in **Table 1** show that the bone volume to total volume ratio (BV/TV) and connectivity density (Conn.Dn) of young female chronically drinking rats were lower than those of the normal group (*p *< 0.05), whereas the trabecular separation (Tb.Sp) in female rats was higher (*p *< 0.05), indicating the occurrence of bone resorption. The structural model index (SMI) of male and female chronically drinking rats was significantly higher than that of the normal group (*p *< 0.001), indicating that the trabecular bone was transformed from a plate-like architecture to a rod-like architecture. Furthermore, chronic alcohol consumption significantly decreased BMD in young female and male rats (*p *< 0.001 and *p *< 0.01, respectively).

**Table 1:** Changes of bone micro-CT quantitative parameters in each group.

Groups	BV/TV (%)	Tb.Sp (mm)	Conn.Dn (1/mm^3^)	SMI	BMD (g/cm^3^)
MN	26.95 ± 1.57	0.23 ± 0.03	99.52 ± 16.97	1.36 ± 0.01	0.31 ± 0.003
MA	24.45 ± 1.19	0.29 ± 0.01	76.06 ± 1.10	1.56 ± 0.04^***^	0.23 ± 0.01^***^
FN	26.13 ± 0.39	0.26 ± 0.01	97.28 ± 15.87	1.37 ± 0.03	0.31 ± 0.01
FA	20.20 ± 4.42^*^	0.39 ± 0.09^*^^,^ ^#^	63.64 ± 23.90^*^	1.65 ± 0.02^***^^,^ ^#^	0.28 ± 0.01^**^^,^ ^###^

MN: male normal rat group, MA: male administration alcohol rat group, FN: female normal rat group, FA: female administration alcohol rat group, BV/TV: Bone Volume/ Total Volume, Tb.Sp: Trabecular Separation, Conn.Dn: Connectivity Density, SMI: Structure model index, BMD: Bone mineral density. Data were analyzed using two-way ANOVA with Bonferroni post-hoc for multiple pairwise comparisons.

* *p* < 0.05,

** *p* < 0.01,

*** *p* < 0.001, compared with control of the same sex,

# *p* < 0.05,

### *p* < 0.001, compared with the MA group.

**Fig 1:**
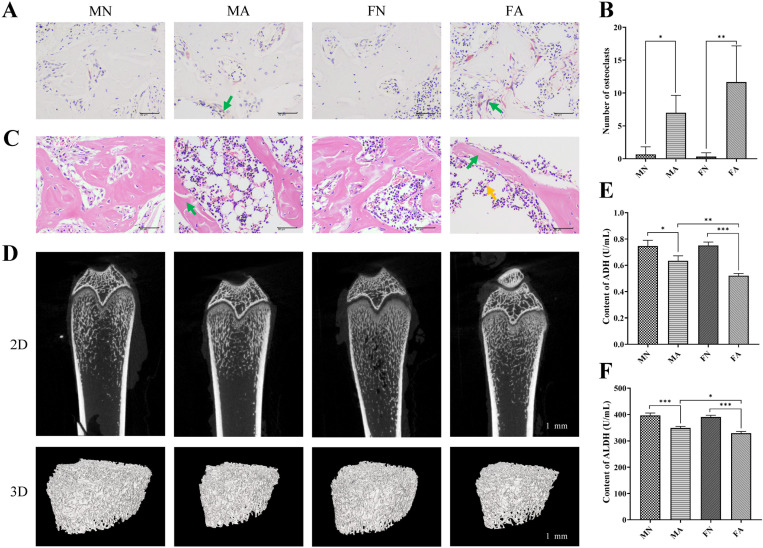
Effects of alcohol on bone morphology and alcohol metabolism ability in female and male rats. (**A**) TRAP staining image of femur with green arrows indicate osteoclasts. Scale bar: 50 μm, 400 × . (**B**) Number of osteoclasts observed with TRAP staining in different groups. Two-way ANOVA of osteoclasts number: sex effect *p* = 0.264, alcohol effect *p* = 0.001, no significant sex-by-alcohol interaction (*p* = 0.203). (**C**) H&E staining image of femur. Green arrows indicate thinning of bone trabecula, yellow arrows indicate decreased bone marrow hematopoietic cells. Scale bar: 50 μm, 400 × . (**D**) 2D (scale bar: 1 mm) and 3D (scale bar: 1 mm) images of femur Micro-CT scan. Changes of alcohol metabolism indexes ADH (**E**) and ALDH (**F**) contents after long-term alcohol intake. Two-way ANOVA of ADH: sex effect *p* = 0.022, alcohol effect *p* = 0.000, with significant sex-by-alcohol interaction (*p* = 0.015). Two-way ANOVA of ALDH: sex effect *p* = 0.019, alcohol effect *p* = 0.000, no significant sex-by-alcohol interaction (*p* = 0.159). Date are shown as mean ± SD (*n* = 6). ^*^p < 0.05, ^**^p < 0.01, ^***^p < 0.001.

### Effect of alcohol metabolism capacity in young female and male rats

In this study, the levels of alcohol dehydrogenase (ADH) and aldehyde dehydrogenase (ALDH) in the blood of male and female rats before and after alcohol consumption were observed to explore the differences in alcohol metabolism between sexes. With regards to ADH, there were significant sex main effects (F = 8.026, *p* = 0.022 < 0.05, η^2^ = 0.501), significant alcohol main effects (F = 80.800, *p* = 0.000 < 0.05, η^2^ = 0.910), and significant sex × alcohol interaction (F = 9.474, *p* = 0.015 < 0.05, η^2^ = 0.542) were found (S2 Table), suggesting that there were interaction between sex and alcohol in ADH levels. With regards to ALDH, there were significant sex main effects (F = 8.684, *p* = 0.019 < 0.05, η^2^ = 0.550) and significant alcohol main effects (F = 163.061, *p* = 0.000 < 0.05, η^2^ = 0.953) were found, but no significant sex × alcohol interaction (F = 2.411, *p* = 0.159 > 0.05, η^2^ = 0.232) (S2 Table). The results of multiple comparisons showed no significant difference between the MN and FN groups, and the levels of ADH and ALDH in the MA and FA groups were significantly lower than those in the normal group (*p* < 0.05, *p* < 0.001, respectively). In addition, the levels of ADH and ALDH were higher in MA rats than in FA rats (*p* < 0.01, *p* < 0.05), indicating that the ethanol metabolic ability of male rats was stronger than that of female rats (**Fig 1E**, **1F**).

### Effects of alcohol on bone metabolism, bone resorption, and serum biochemical indicators in young female and male rats

**Fig 2:**
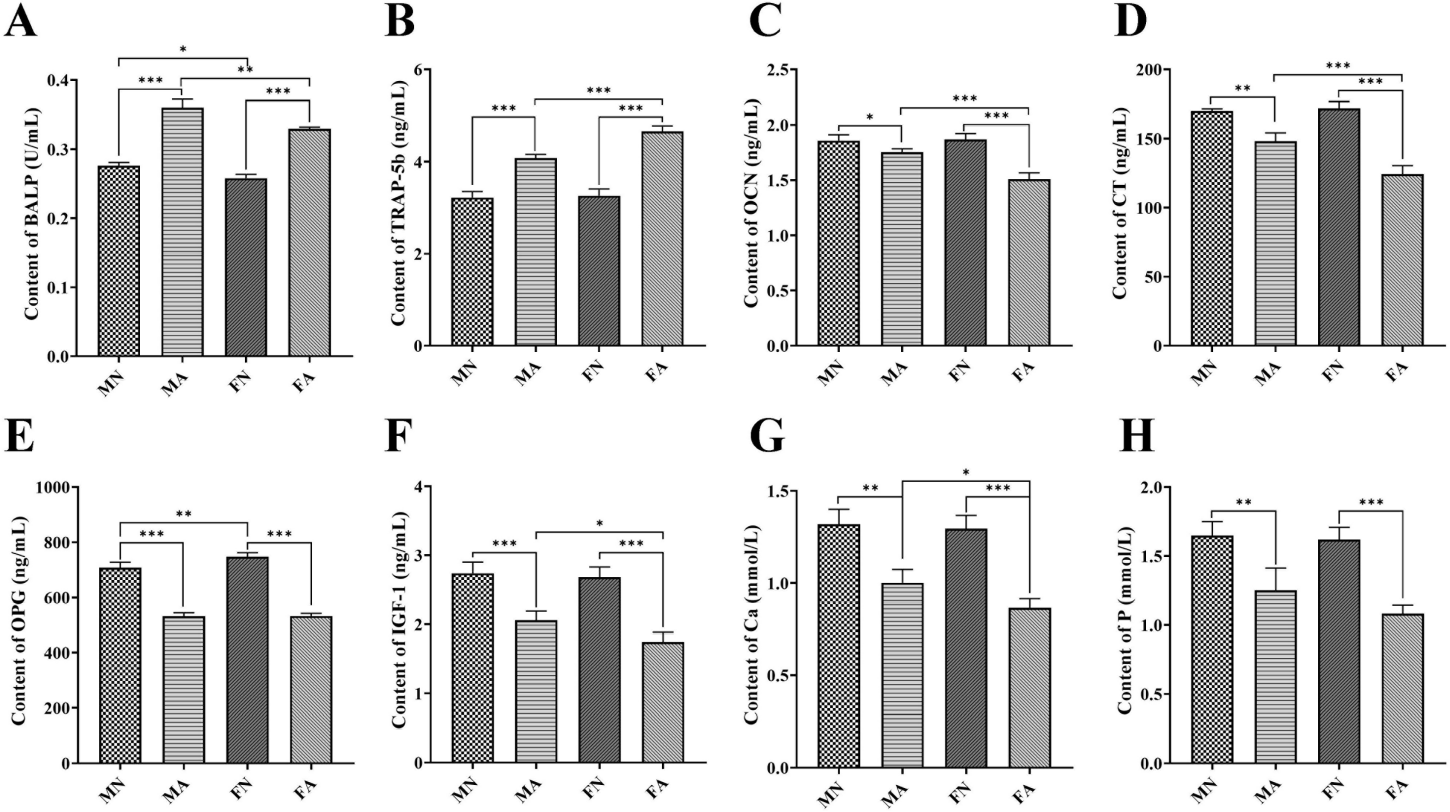
Effect of alcohol on bone resorption and bone formation in female and male rats. (**A**) Changes of BALP levels in different groups. Two-way ANOVA of BALP: sex effect *p* = 0.000, alcohol effect *p* = 0.000, no significant sex-by-alcohol interaction (*p* = 0.169). (**B**) Changes of TRAP-5b levels in different groups. The levels of TRAP-5b are commonly used in the evaluation of bone resorption. Two-way ANOVA of TRAP-5b: sex effect *p* = 0.002, alcohol effect *p* = 0.000, with significant sex-by-alcohol interaction (*p* = 0.005). (**C**) Long-term alcohol consumption decreased OCN levels in male and female rats, indicating reduced bone formation in the femur. Two-way ANOVA of OCN: sex effect *p* = 0.004, alcohol effect *p* = 0.000, with significant sex-by-alcohol interaction (*p* = 0.002). (**D**) The CT levels decreased after drinking, indicating increased osteoclast activity. Two-way ANOVA of CT: sex effect *p* = 0.005, alcohol effect *p* = 0.000, with significant sex-by-alcohol interaction (*p* = 0.002). (**E**) The levels of OPG decreased after long-term alcohol consumption, indicating enhanced bone resorption. Two-way ANOVA of OPG: sex effect *p* = 0.038, alcohol effect *p* = 0.000, with significant sex-by-alcohol interaction (*p* = 0.047). (**F**) The levels of IGF-1 were detected by ELISA. Two-way ANOVA of IGF-1: sex effect *p* = 0.057, alcohol effect *p* = 0.000, no significant sex-by-alcohol interaction (*p* = 0.162). The contents of Ca (**G**) and P (**H**) also decreased with alcohol intake. Two-way ANOVA of Ca: sex effect *p* = 0.086, alcohol effect *p* = 0.000, no significant sex-by-alcohol interaction (*p* = 0.205). Two-way ANOVA of P: sex effect *p* = 0.159, alcohol effect *p* = 0.000, no significant sex-by-alcohol interaction (*p* = 0.309). Date are shown as mean ± SD (*n* = 6). ^*^p < 0.05, ^**^p < 0.01, ^***^p < 0.001.

With regards to BALP, there were significant sex main effects (F = 36.015, *p* = 0.000 < 0.05, η^2^ = 0.828) and alcohol main effects (F = 363.482, *p* = 0.000 < 0.05, η^2^ = 0.978), but no significant sex × alcohol interaction (F = 2.282, *p* = 0.169 > 0.05, η^2^ = 0.222) (S3 Table). The BALP levels (**Fig 2A**) in male and female alcohol-treated rats were significantly higher than those in the control group, indicating that long-term alcohol consumption can lead to liver function impairment and increased osteoblastic activity. With regards to TRAP-5b, there were significant sex main effects (F = 19.432, *p* = 0.002 < 0.05, η^2^ = 0.708), alcohol main effects (F = 256.629, *p* = 0.000 < 0.05, η^2^ = 0.970), and sex × alcohol interaction (F = 14.461, *p* = 0.005 < 0.05, η^2^ = 0.644) (S3 Table). The levels of TRAP-5b (**Fig 2B**) in male and female alcohol-treated rats were significantly higher than those in the control group, indicating that chronic alcohol consumption can lead to bone resorption. Furthermore, BALP levels were higher in the MA group than those in the FA group (*p* < 0.01), whereas TRAP-5b levels were lower in the MA group than those in the FA group (*p* < 0.001). There were significant main effect of sex and alcohol for OCN (sex main effect, F = 16.074, *p* = 0.004 < 0.05, η^2^ = 0.668; alcohol main effect, F = 65.225, *p* = 0.000 < 0.05, η^2^ = 0.891; sex × alcohol interaction, F = 19.787, *p* = 0.002 < 0.05, η^2^ = 0.712), CT (sex main effect, F = 14.512, *p* = 0.005 < 0.05, η^2^ = 0.645; alcohol main effect, F = 144.351, *p* = 0.000 < 0.05, η^2^ = 0.947; sex × alcohol interaction, F = 19.701, *p* = 0.002 < 0.05, η^2^ = 0.711), and OPG (sex main effect, F = 6.139, *p* = 0.038 < 0.05, η^2^ = 0.434; alcohol main effect, F = 539.025, *p* = 0.000 < 0.05, η^2^ = 0.985; sex × alcohol interaction, F = 5.502, *p* = 0.047 < 0.05, η^2^ = 0.407) levels (S3 Table). After long-term alcohol consumption, the levels of OCN (**Fig 2C**), CT (**Fig 2D**), and OPG (**Fig 2E**) in the serum of male and female alcohol-treated rats were significantly lower than those in the control group (both *p* < 0.05). In addition, OCN and CT levels were higher in the MA group than in the FA group (both *p* < 0.001). In addition, there were significant alcohol main effect for IGF-1 (F = 90.748, *p* = 0.000 < 0.05, η^2^ = 0.919), Ca (F = 86.056, *p* = 0.000 < 0.05, η^2^ = 0.915), and P (F = 54.113, *p* = 0.000 < 0.05, η^2^ = 0.871) levels, whereas there were no significant sex main effect and interaction effect (S3 Table). The levels of IGF-1 (**Fig 2F**), Ca (**Fig 2G**), and P (**Fig 2H**) in the MA and FA groups were significantly lower than those in the normal group (both *p* < 0.01). Furthermore, IGF-1 and calcium levels were significantly lower in the FA group than in the MA group (*p* < 0.05). In summary, the interaction between sex and alcohol consumption had a significant effect on TRAP-5b, OCN, CT, and OPG levels.

### Effect of alcohol on bone marrow immunoreactivity in young female and male rats

**Fig 3:**
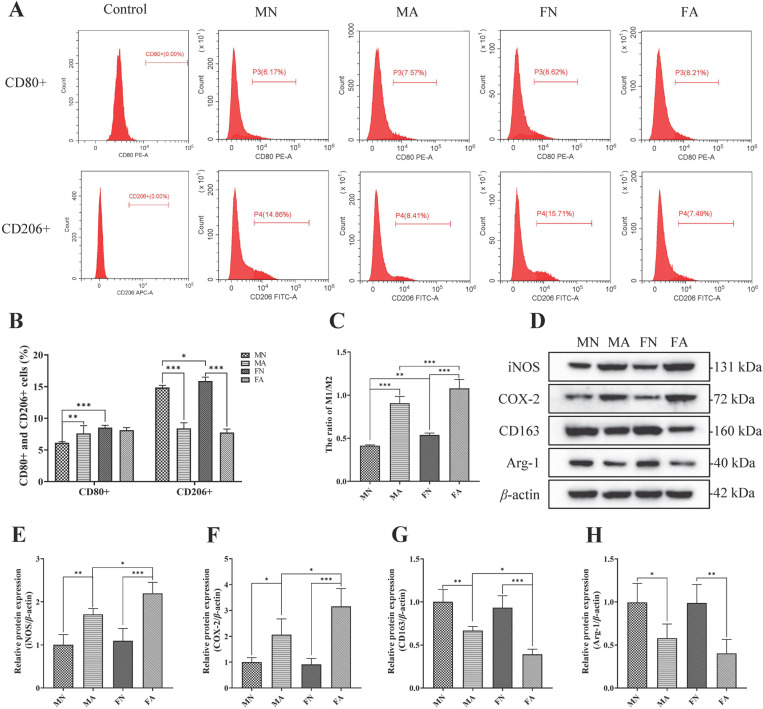
Effect of alcohol on bone marrow immune activity in female and male rats. (**A**) The proportion of CD80 + and CD206 + cells in the bone marrow was analyzed by flow cytometry. (**B**) The numbers of CD80 + cells were significantly higher in the male and female alcohol-treated groups than in the normal group; while the numbers of CD206 + cells were significantly lower. Two-way ANOVA of CD80 + : sex effect *p* = 0.000, alcohol effect *p* = 0.065, with significant sex-by-alcohol interaction (*p* = 0.002). Two-way ANOVA of CD206 + : sex effect *p* = 0.464, alcohol effect *p* = 0.065, with significant sex-by-alcohol interaction (*p* = 0.004). (**C**) Quantitative analysis of the ratio of CD80 + /CD206 + . Two-way ANOVA of CD80 + /CD206 + : sex effect *p* = 0.000, alcohol effect *p* = 0.000, no significant sex-by-alcohol interaction (*p* = 0.382). (**D**) The protein expression of polarization markers of M1/M2 macrophages in bone marrow was detected by WB analysis. (**E-H**) The quantification of WB analysis of iNOS, COX-2, CD163, and Arg-1. Two-way ANOVA of iNOS: sex effect *p* = 0.065, alcohol effect *p* = 0.000, no significant sex-by-alcohol interaction (*p* = 0.182). Two-way ANOVA of COX-2: sex effect *p* = 0.105, alcohol effect *p* = 0.000, no significant sex-by-alcohol interaction (*p* = 0.067). Two-way ANOVA of CD163: sex effect *p* = 0.023, alcohol effect *p* = 0.000, no significant sex-by-alcohol interaction (*p* = 0.129). Two-way ANOVA of Arg-1: sex effect *p* = 0.429, alcohol effect *p* = 0.002, no significant sex-by-alcohol interaction (*p* = 0.467). Date are shown as mean ± SD (*n* = 6). ^*^p < 0.05, ^**^p < 0.01, ^***^p < 0.001.

The number of CD80+ (M1 polarization marker) and CD206+ (M2 polarization marker) cells in the bone marrow was analyzed using flow cytometry (**Fig 3A**). With regards to CD80 + , there were significant sex main effects (F = 27.900, *p* = 0.000 < 0.05, η^2^ = 0.582) and sex × alcohol interaction (F = 12.037, *p* = 0.002 < 0.05, η^2^ = 0.376), but no significant alcohol main effects (F = 3.826, *p* = 0.065 > 0.05, η^2^ = 0.161) (S4 Table). With regards to CD206 + , there were significant alcohol main effects (F = 794.105, *p* = 0.000 < 0.05, η^2^ = 0.975) and sex × alcohol interaction (F = 10.450, *p* = 0.004 < 0.05, η^2^ = 0.343), but no significant sex main effects (F = 0.558, *p* = 0.464 > 0.05, η^2^ = 0.027) (S4 Table). The results showed that the number of CD80 + cells was significantly higher in the male alcohol-treated group than in the normal group (*p *< 0.01), whereas the number of CD206 + cells was significantly lower in male and female alcohol-treated rats than in normal rats (*p *< 0.001) (**Fig 3B**). In addition, the CD80 + /CD206 + ratio was significantly higher in the MA and FA groups than in the MN and FN groups (*p *< 0.001) (**Fig 3C**). There were significant sex main effects (F = 29.718, *p* = 0.000 < 0.05, η^2^ = 0.598) and alcohol main effects (F = 363.461, *p* = 0.000 < 0.05, η^2^ = 0.948), but no significant statistical interaction between sex and alcohol (F = 0.798, *p* = 0.383 > 0.05, η^2^ = 0.038) (S4 Table). To further study the effect of alcohol on the immune activity of rats, the expression levels of iNOS and COX-2 (M1 polarization markers), CD163, and Arg-1 (M2 polarization markers) in the bone marrow were detected by WB blotting (**Fig 3D**). There were significant alcohol main effect for iNOS (F = 44.342, *p* = 0.000 < 0.05, η^2^ = 0.847), COX-2 (F = 35.709, *p* = 0.000 < 0.05, η^2^ = 0.817), CD163 (F = 50.783, *p* = 0.000 < 0.05, η^2^ = 0.864) and Arg-1 (F = 20.394, *p* = 0.000 < 0.05, η^2^ = 0.718), whereas there were no significant sex main effect and interaction effect, except sex main effect for CD163 (F = 7.949, *p* = 0.023 < 0.05, η^2^ = 0.498) (S4 Table). The results showed that The protein expression levels of iNOS (**Fig 3E**) and COX-2 (**Fig 3F**) were higher in the MA and FA groups than those in the MN and FN groups (both *p* < 0.05), and the expression levels of iNOS and COX-2 were higher in the FA group than those in the MA group (*p* < 0.05). The expression levels of CD163 (**Fig 3G**) and Arg-1 (**Fig 3H**) in male and female alcohol-treated rats significantly decreased (both *p* < 0.05), and the expression levels of CD163 in female rats were lower in the FA group than in the MA group (*p* < 0.05). The original western blot image is shown as S1_RAW_IMAGES. These results indicated that although alcohol had an effect on bone marrow immune activity in rats, there was no interaction between sex and alcohol consumption.

### Effect of alcohol on gut microbiota diversity in young female and male rats

**Fig 4:**
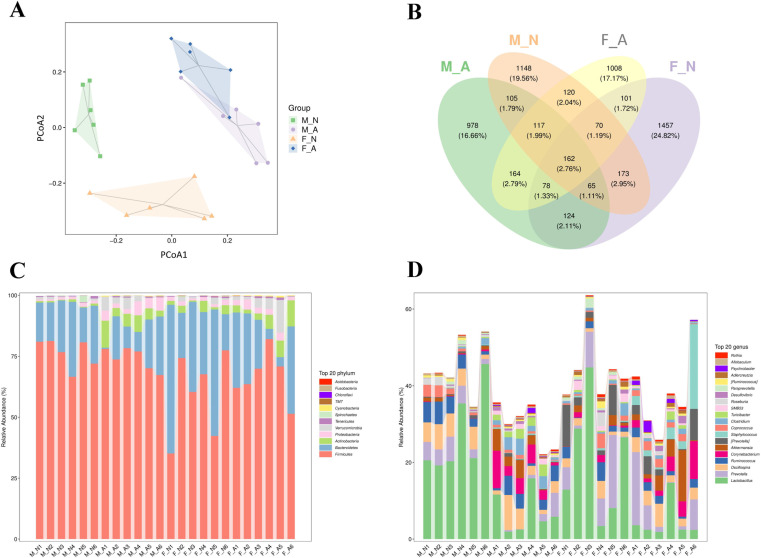
Effect of alcohol on gut microflora in female and male rats. (**A**) PCoA analysis of beta diversity by Bray-Curtis distance. (**B**) Venn diagram of ASV/OTU of samples. (**C**) The composition of the intestinal microbiota of all samples at the phylum levels. (**D**) The composition of the intestinal microbiota at the genus level. *n* = 6. ASV: amplicon sequence variants; OUT: operational taxonomic units.

To investigate the effects of chronic alcohol consumption on the gut microbiota of young male and female rats, 16S rRNA sequencing was used to analyze gut microbiota diversity in rat feces. Beta diversity was analyzed by PCoA to assess the differences between samples (**Fig 4A**). The greater the distance between two points, the greater the difference in the microbial communities between the two samples. The results showed that male and female rats with long-term alcohol consumption were far from the normal group, indicating that long-term alcohol consumption significantly altered microbial diversity. The Venn graph showed 162 common operational taxonomic units (OTUs) in all groups, and the numbers of unique OTUs in the MN, MA, FN, and FA groups were 1148, 978, 1457, and 1008, respectively (**Fig 4B**). These OTUs may indicate changes in intestinal microbiota after long-term alcohol consumption.

To further explore the composition and distribution of species, changes in their relative abundance were analyzed at the phylum (**Fig 4C**) and genus levels (**Fig 4D**). The results showed that at the phylum level, the highest relative abundance was found for *Firmicutes*, *Bacteroidetes*, *Actinobacteria*, *Proteobacteria*, *Verrucomicrobia*, *Tenericutes*, *Spirochaetes*, *Cyanobacteria*, *TM7*, *Chloroflexi* and *Fusobacteria*. After long-term alcohol consumption, the ratio of Firmicutes/Bacteroidetes decreased significantly as a consequence of a greater abundance of Bacteroidetes and lesser of Firmicutes (*p* < 0.05) (S1 Fig A). In addition, long-term alcohol consumption increased the relative abundance of *Actinobacteria*, *Proteobacteria*, *Verrucomicrobia*, and *Cyanobacteria*. At the genus level, the genera with high relative abundances were *Lactobacillus*, *Prevotella*, *Oscillospira*, *Ruminococcus*, *Corynebacterium*, *Akkermansia*, [*Prevotella*], *Staphylococcus*, *Coprococcus*, *Clostridium*, *etc* (S1 Fig B). In addition, compared with the MN and FN groups, the MA and FA groups had significantly fewer *Lactobacillus.*

### Effects of alcohol on fecal metabolites in young female and male rats

**Fig 5:**
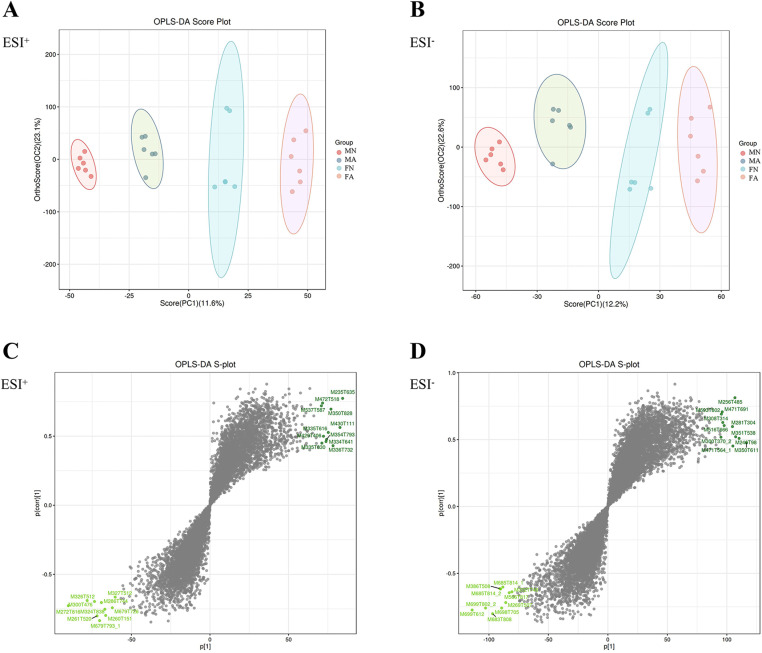
Multivariate statistical analysis of gut microbiota metabolites data in positive and negative ion mode. (**A-B**) OPLS-DA analysis in positive and negative ion mode. The more clustered the samples within groups, the more dispersed the samples between groups, indicating more reliable results. (**C-D**) OPLS-DA score plot in positive and negative ion mode. Metabolites closer to two corners are more important.

**Fig 6:**
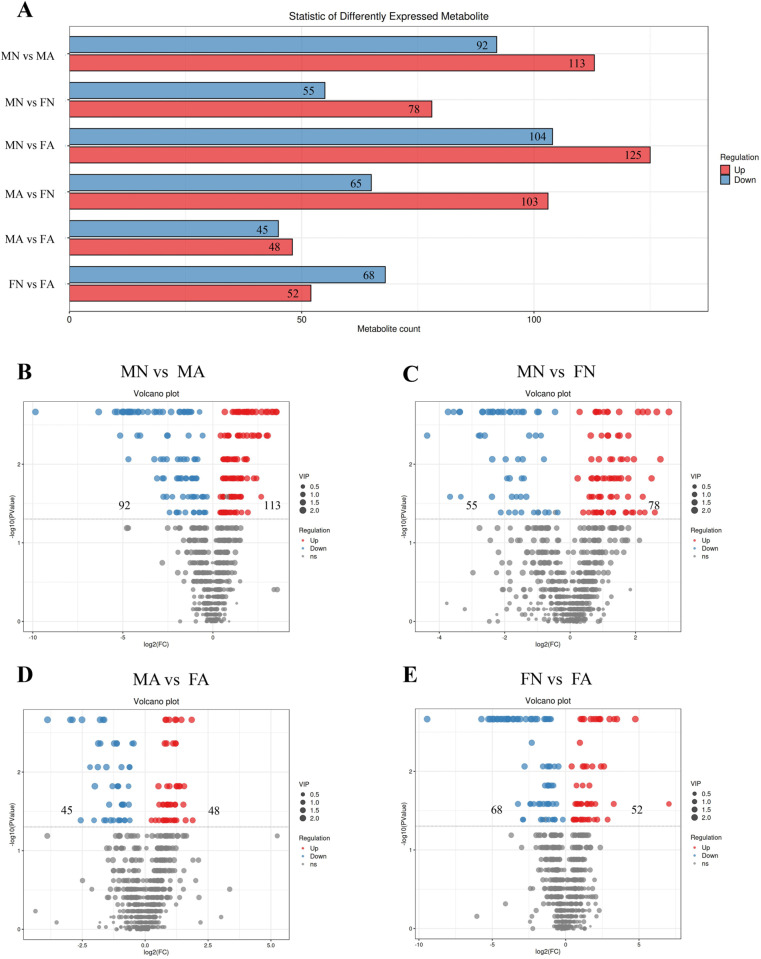
Identification of fecal differential metabolites in each group. (**A**) Statistical bar chart of different metabolites in MN *vs* MA, MN *vs* FN, MA *vs* FA, FN *vs* FA, respectively. (**B-E**) Volcano plot of differential metabolites in MN *vs* MA groups, MN *vs* FN groups, MA *vs* FA groups, FN *vs* FA groups, respectively. Red dots represent up-regulated differentially expressed metabolites and blue dots represent down-regulated differentially expressed metabolites.

**Fig 7:**
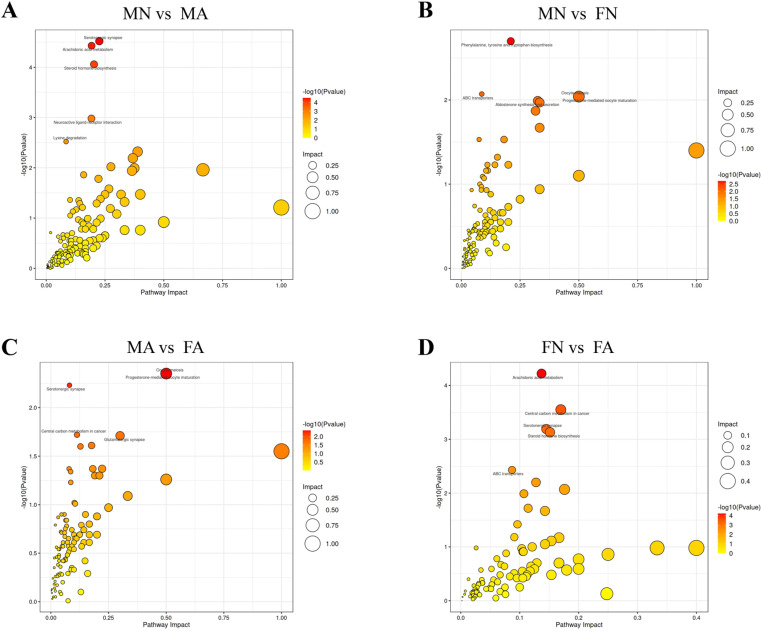
Bubble diagram of differential metabolic pathway influencing factors. (**A**) MN *vs* MA groups; (**B**) MN *vs* FN groups; (**C**) MA *vs* FA groups; (**D**) FN *vs* FA groups. P-value represents the effect of detected differential metabolites on this pathway, and impact represents the contribution of metabolites detected under this pathway.

UPLC-QTOF/MS was used for fecal sample separation and data collection to obtain the base peak chromatogram (BPC) of the MN, MA, FN, and FA groups in positive and negative ion modes, as shown in S2 Fig A, B. The OPLS-DA substitution test showed that OPLS-DA achieved high separation ability, and the results were reliable and effective (S2 Fig C). The model prediction parameters R2Y were greater than 0.9, and Q2 was greater than 0.7, indicating that OPLS-DA had strong reliability and predictive ability (S2 Fig D). The results of the orthogonal projections to latent structures discriminant analysis (OPLS-DA) showed that the MN, MA, FN, and FA groups tended to separate significantly in the positive and negative ion modes, indicating that the fecal metabolism of drinking rats was different from that of non-drinking rats (**Fig 5A**, B). According to the variable importance for the projection (VIP) values and the S-plot graph, metabolites with strong correlations with major components in biological processes were screened (**Fig 5C**, **5D**). Differential metabolites with VIP > 1 and *p* < 0.05 were selected as potential biomarkers, and a total of 754 differential metabolites were obtained by high-precision molecular ion peaks detected by MS/MS fragment information (S5 Table). In total, 205 differential metabolites were identified between the MN and MA groups, of which 113 were significantly upregulated and 92 were significantly downregulated. In the MN and FN groups, 133 differential metabolites were identified, of which 78 were significantly upregulated, and 55 were significantly downregulated. Ninety-three differential metabolites were identified in the MA and FA groups, 93 differential metabolites were identified, of which 48 were upregulated and 45 were downregulated. In total, 120 differential metabolites were identified in the FN and FA groups, of which 52 were upregulated and 68 were downregulated (**Fig 6A**). Differential metabolite classification is associated with hormones such as androsterone, etiocholanolone, hydroxypregnenolone, prostaglandin, short-chain fatty acids, amino acids, and some steroids. The amounts and trends of metabolites in the different groups are shown in the volcano diagram (**Fig 6B**–**6E**). From KEGG analysis, it was seen that metabolic differences between MN and MA groups were mainly associated with serotonergic synapse, arachidonic acid metabolism, and arachidonic acid metabolism (**Fig 7A**). The MN and FN groups were primarily associated with phenylalanine, tyrosine, and tryptophan biosynthesis (**Fig 7B**). The metabolic differences between the MA and FA groups were mainly associated with oocyte-mediated meiosis and progesterone-oocyte maturation (**Fig 7C**). The FN and FA groups were primarily involved in arachidonic acid metabolism, central carbon metabolism in cancer, serotonergic synapses, and steroid hormone biosynthesis (**Fig 7D**). The results showed that chronic alcohol consumption led to a disturbance in fecal metabolites and affected multiple metabolic pathways.

## Discussion

Over the last few decades, increasing evidence has shown the deleterious effects of chronic alcohol consumption on bone health. Moreover, the study showed that male Chinese adolescents had higher drinking frequency, alcohol consumption, heavy drinking, and drinking-related problems than female adolescents. Alcohol consumption of more than four glasses per day in men and two glasses per day in women is a major risk factor for osteoporosis and fractures, suggesting that alcohol consumption may have different effects on sex [26]. Alcohol consumption leads to decreased bone mineral density, osteonecrosis, and trabecular rupture, and greatly increases the risk of fracture [27,28]. Alcohol affects osteoblasts and osteoclasts as well as other pathways such as protein metabolism, liver function, and endocrine function to disrupt bone metabolism homeostasis [29,30]. In clinical examinations, bone mineral density (BMD) is an important diagnostic index for evaluating bone strength. In this study, we found that the number of osteoclasts increased, and bone mineral density decreased in rats after chronic alcohol consumption. Alcohol metabolism relies mainly on ADH and ALDH, and alcohol is catabolized to acetaldehyde and acetic acid by ADH and ALDH [31]. The results of this study showed that ADH and ALDH levels in rats with long-term alcohol consumption were significantly lower than those in the control group, and those in male rats were higher than those in female rats. This indicates that male rats have a higher capacity to metabolize alcohol than female rats do. Age-related bone loss is caused by an imbalance in bone remodeling, that is, an imbalance in bone formation and resorption [32]. Bone loss is exacerbated when osteoclast hyperactivation leads to enhanced bone resorption and osteoblast inhibition leads to reduced bone formation [33]. BALP is an important marker of liver function and osteoblast activity [34]. Chronic alcohol consumption results in increased BALP levels, indicating liver injury and increased osteoblast activity caused by alcohol. TRAP-5b is an important marker of bone resorption. Our study showed that alcohol increased the levels of TRAP-5b, but TRAP-5b levels were more pronounced in females. This suggests that alcohol may predispose females to bone resorption, which is consistent with the clinical manifestations [35]. OCN is an osteoblast-specific protein that is considered an important marker of bone formation [36]. CT regulates Ca and P metabolism and inhibits osteoclast activity [37]. OPG levels negatively correlate with bone resorption [38]. IGF-1 is an abundant growth factor in the bone that inhibits bone resorption and promotes bone formation [39]. Our study showed that long-term alcohol consumption decreased bone formation and enhanced bone resorption in young male and female rats, resulting in femoral decalcification. In addition, studies have reported that many alcoholics have high chronic stress, and that the frequency of alcohol consumption in alcoholics is positively correlated with stress levels. It is worth noting that gavage is a sufficiently strong stressor to potentially induce bone resorption. One limitation of this study was the lack of investigation into the influence of stress caused by oral gavage on bone resorption. Notably, this study examined only the effects of 12 weeks of alcohol treatment in young male and female rats, and did not discuss the long-term effects of alcohol consumption in young male and these rats.

The immune system plays a crucial role in the maintenance of bone formation and resorption [40]. Moreover, our previous studies demonstrated that chronic alcohol consumption promotes NF-*κ*B ligand receptor-activating factor (RANKL) signaling and inflammatory responses in the immune system, further promoting excessive osteoclast activation [9]. The balance between M1 and M2 macrophage polarization is closely associated with bone remodeling [41]. When M1/M2 macrophages are imbalanced, T cell-specific cytokines induce the overexpression of RANKL, ultimately triggering inflammatory and osteoporotic processes [24]. Macrophage-associated surface markers such as CD80, CD163, and CD206 are often used to identify macrophage phenotypes and play an important role in inhibiting the inflammatory response, identifying pathogens, and antigen presentation [42]. In this study, alcohol significantly increased the ratio of M1/M2 polarization, suggesting that alcohol promotes polarization toward M1 in young male and female rats and that there was a significant sex and alcohol interaction. M1 macrophages promote the occurrence of osteoclasts through reactive oxygen species (ROS) and inflammatory factors, whereas M2 macrophages have osteoprotective effects [43]. Studies have shown that Arg-1 is a negative regulator of osteoclast differentiation, and excessive osteoclast differentiation decreases Arg-1 levels [44]. In this study, alcohol increased the polarization of the macrophage-induced inflammation (M1) phenotype, as indicated by increased iNOS and COX-2 protein levels. However, alcohol significantly decreased the expression of CD163 and Arg-1 (M2 polarization markers), indicating that long-term alcohol consumption led to osteoclast differentiation. In addition, the protein levels of iNOS and COX-2 in female rats with long-term alcohol consumption were significantly higher than those in male rats, and the expression levels of CD163 and Arg-1 were significantly lower than those in male rats, indicating that alcohol is more likely to cause bone resorption in female rats. Our findings demonstrate that chronic alcohol consumption affects immune activity in young male and female rats. However, the relationship between immunity and inflammation requires further investigations.

Gut microbiota is a complex intestinal biological community that maintains the intestinal microenvironment. Some studies have demonstrated a close association between gut microbiota and bone health [45]. Intestinal microecology is an important factor regulating bone metabolism [46]. Excessive alcohol consumption changes the microbial structure and the intestinal environment of the body, thereby destroying its microbiology. At the same time, it can also cause oxidative stress, immune and inflammatory reactions, and bone metabolism disorders [47,48]. In this study, 16S rRNA sequencing was used to analyze the effects of chronic alcohol consumption on the gut microbiota of young male and female rats. Analysis of *β*-diversity showed that chronic alcohol consumption rats could be clearly distinguished from normal rats, indicating that the intestinal microbiota diversity of alcohol-treated rats was changed. At the phylum level, the dominant intestinal bacteria were mainly *Firmicutes*, *Bacteroidetes*, *Actinobacteria*, *Proteobacteria*, *Verrucomicrobia*, *Tenericutes*, *Spirochaetes*, *Cyanobacteria*, *TM7*, *Chloroflexi*, and *Fusobacteria*. In this study, it was found that the ratio of *Firmicutes*/*Bacteroides* decreased after chronic alcohol consumption. Previous studies have shown that the ratio of *Firmicutes* to *Bacteroidetes* negatively correlates with osteoclast differentiation [49], indicating that chronic alcohol consumption aggravates osteoclast differentiation in male and female rats. At the genus level, *Lactobacillus*, *Prevotella*, *Oscillospira*, *Ruminococcus*, *Corynebacterium*, *Akkermansia*, [*Prevotella*], *Staphylococcus*, *Coprococcus*, and *Clostridium* accounted for relatively large proportions. In addition, *Lactobacillus*, a beneficial gut microbe, has beneficial anti-inflammatory properties and can prevent bone loss and increase bone density [50]. These results also suggest that chronic alcohol consumption may lead to a significant decrease in the proportion of *Lactobacillus*.

Untargeted metabolomics contributes to the investigation of the effects of chronic alcohol consumption on metabolite differences between male and female rats. The metabolomics of fecal samples were analyzed using UPLC-TOF-MS/MS. We identified 205 differential metabolites in the MN and MA groups: 133 in the MN and FN groups, 93 in the MA and FA groups, and 120 in the FN and FA groups. To further clarify the differential metabolic pathways, we performed a KEGG pathway analysis. The results showed that serotonergic synapses, arachidonic acid metabolism, phenylalanine, tyrosine, tryptophan biosynthesis, omeocyte, progesterone-oocyte mediated maturation, central carbon metabolism in cancer, and steroid hormone biosynthesis pathways were significantly correlated. Studies have shown that 5-HT participates in communication between osteocytes and other organs, regulates bone homeostasis, inhibits osteoblast differentiation, and increases osteoclast activation [51]. Arachidonic acid and unsaturated fatty acids enhance osteoclast production [52]. Phenylalanine, tyrosine, and tryptophan biosynthetic pathways stimulate osteoclast generation, resulting in bone remodeling imbalances and bone fragility [53]. In addition, steroid hormone biosynthesis pathways regulate estrogen and androgens to maintain bone mineral density in osteoporosis [54]. Therefore, alcohol may affect sex hormone levels and regulate bone metabolism. Our results suggest that chronic alcohol consumption contributes to bone loss by inhibiting osteoblast formation and enhancing osteoclast activity by affecting fecal metabolites in young male and female rats. However, the relationships between fecal metabolites and inflammation, immunity, and intestinal flora require further investigation. Future studies using an antibiotic cocktail are needed to further clarify the effects of the gut flora on young male and female rats.

## Conclusions

This study showed that long-term alcohol consumption affects bone formation, induces hyperactivation of bone resorption, and reduces bone mineral density in young male and female rats. Furthermore, chronic alcohol consumption increases bone marrow immune activity, resulting in an imbalance in M1/M2 macrophages and aggravation of osteoclast differentiation. Alcohol also reduces the diversity of intestinal microbiota, affects its structure, and results in variations in fecal metabolites. In addition, the effects of alcohol differed between the young male and female rats. Male rats have a stronger ability to metabolize alcohol than female rats. However, female rats have a lower ability to modulate immunity and a lower diversity of gut microbiota, making alcohol more likely to cause bone damage in female rats.

## Supporting information

S1 Fig The relative abundance of intestinal microbiota. (A) The relative abundance of gut microbiota at the phylum level after long-term alcohol consumption. (B) Relative abundance of at the genus level. Data were shown as mean ± SD, *n *= 6. ^*^p < 0.05, ^**^p < 0.01, compared with control of the same sex; ^#^*p* < 0.05, compared with the different sex.
(TIF)

S2 Fig Untargeted metabolomics was used to analyze the differences of fecal metabolites in rats. (A) Sample base peak chromatogram (BPC) in positive ion mode. (B) Sample BPC in negative ion mode. (C) OPLS-DA permutation test of MN, MA, FN, FA groups. (D) OPLS-DA model validation parameters.
(TIF)

S1 Table The detection limits, and CV of intra- and inter-assays of used ELISA kits. (DOCX)

S2 Table Two-way ANOVA analysis of Figure 1. (DOCX)

S3 Table Two-way ANOVA analysis of Figure 2. (DOCX)

S4 Table Two-way ANOVA analysis of Figure 3. (DOCX)

S5 Table The information of 754 differential metabolites in UPLC-QTOF/MS analysis. (XLSX)

S1 Raw Images The original western blot images involved in the study. (PDF)
