# Supplementary figures and images for: Sex differences in alcohol inhibits bone formation and promotes bone resorption in young male and female rats by altering intestinal flora, metabolites, and bone microenvironment
Source: PLoS One. 2025 May 8;20(5):e0323222. doi: 10.1371/journal.pone.0323222 (PMC12061194; doi:10.1371/journal.pone.0323222)

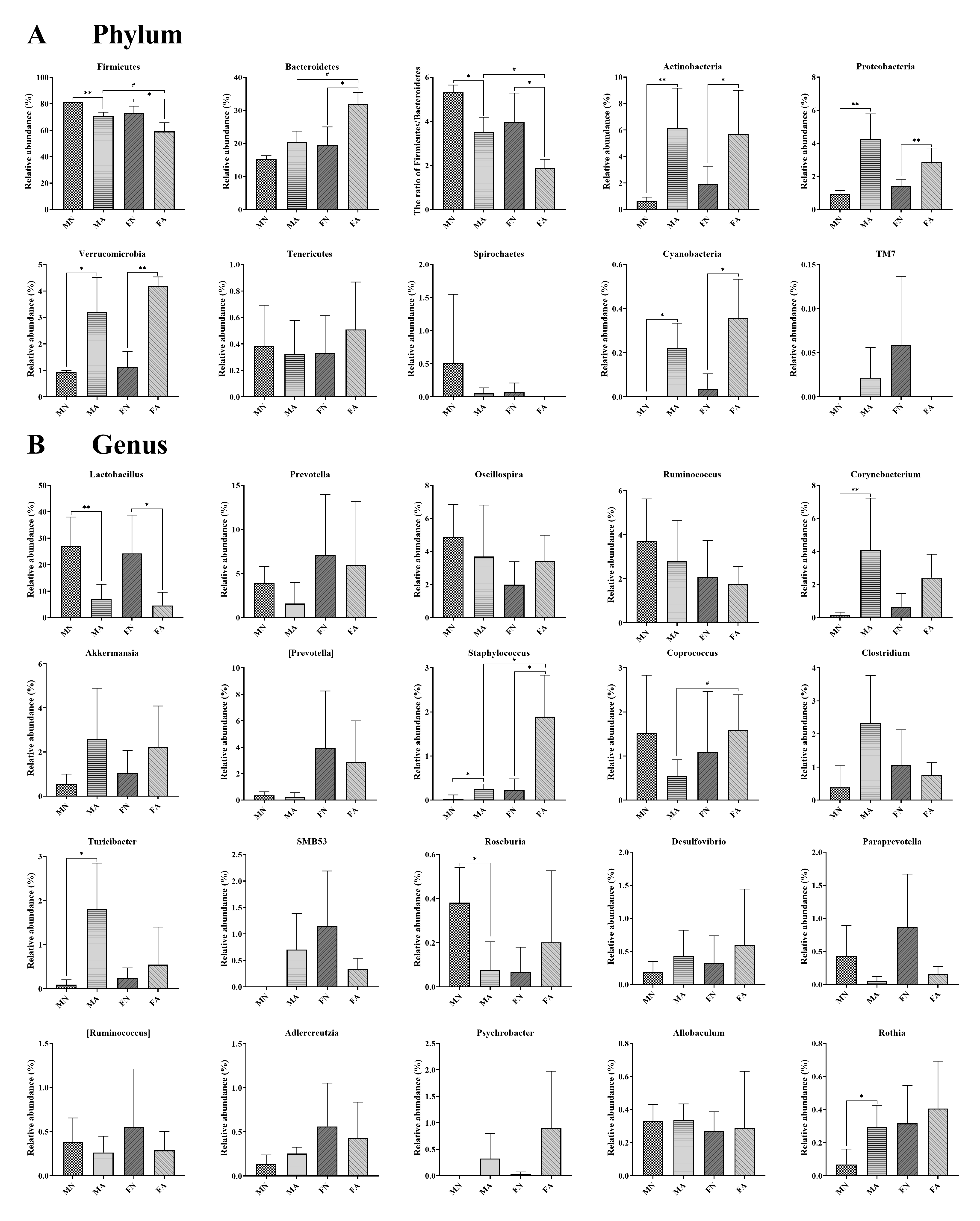

Supplement: S1 Fig — (A) The relative abundance of gut microbiota at the phylum level after long-term alcohol consumption. (B) Relative abundance of at the genus level. Data were shown as mean ± SD, n = 6. *p < 0.05, **p < 0.01, compared with control of the same sex; #p < 0.05, compared with the different sex. (TIF) [file pone.0323222.s001.tif]

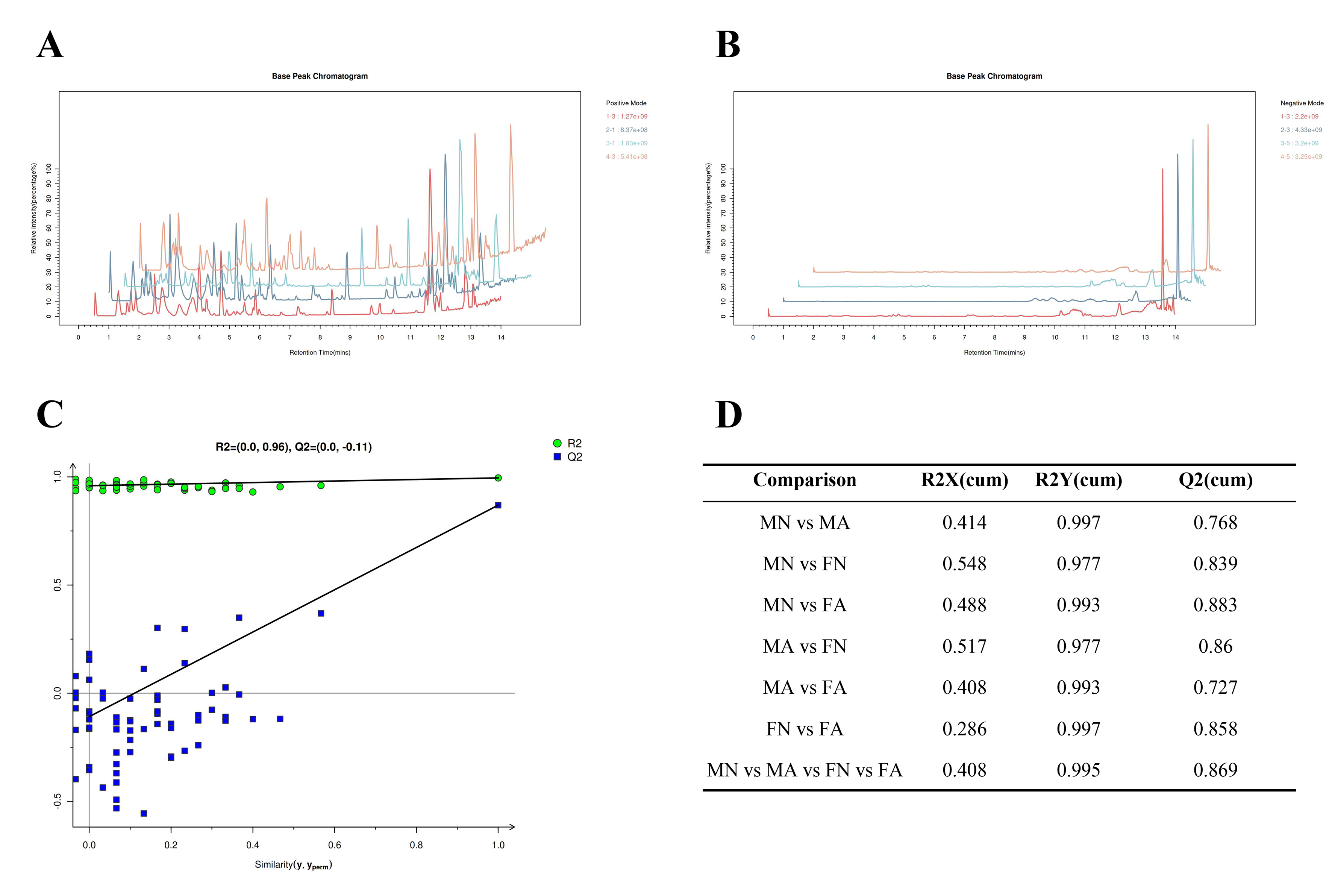

Supplement: S2 Fig — (A) Sample base peak chromatogram (BPC) in positive ion mode. (B) Sample BPC in negative ion mode. (C) OPLS-DA permutation test of MN, MA, FN, FA groups. (D) OPLS-DA model validation parameters. (TIF) [file pone.0323222.s002.tif]

Original image of Western blot

Arg-1

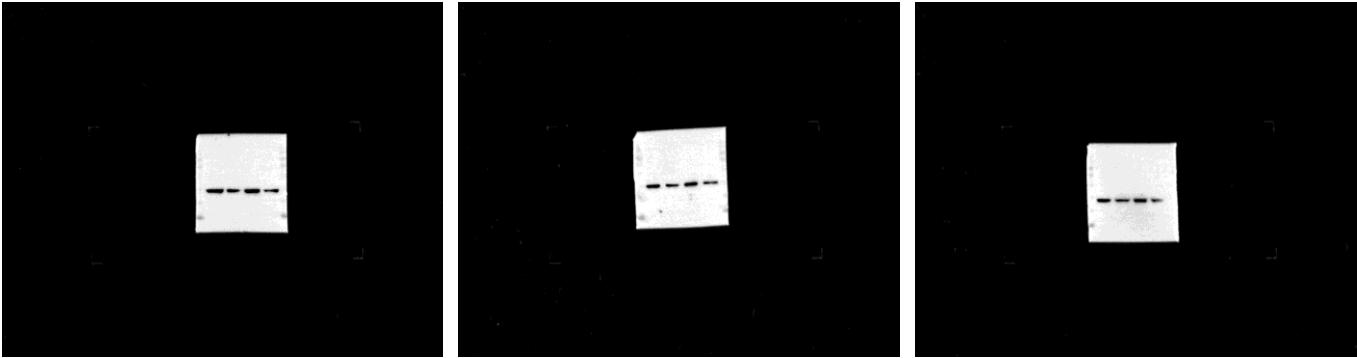

CD163

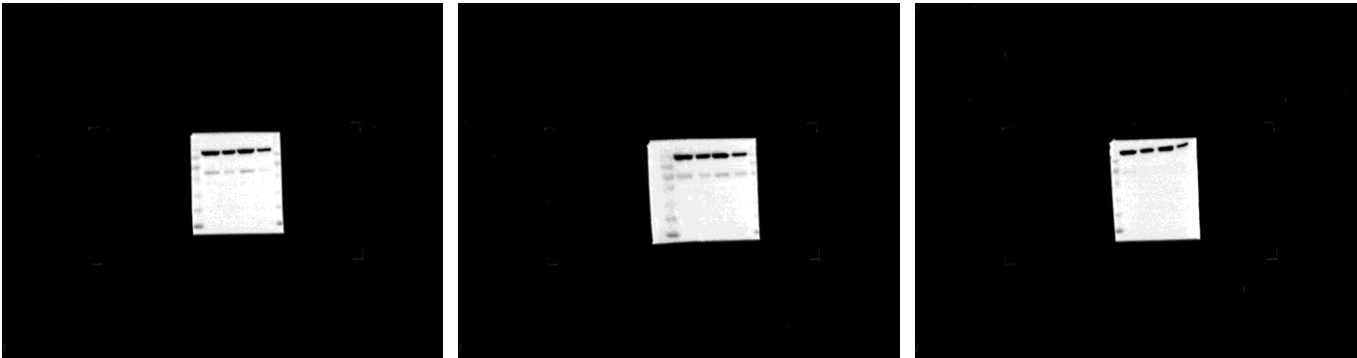

COX2

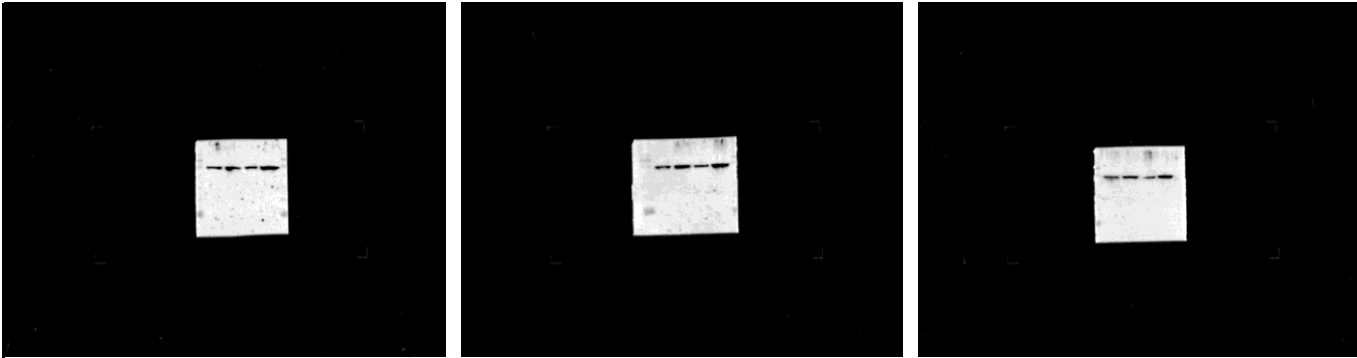

iNOS

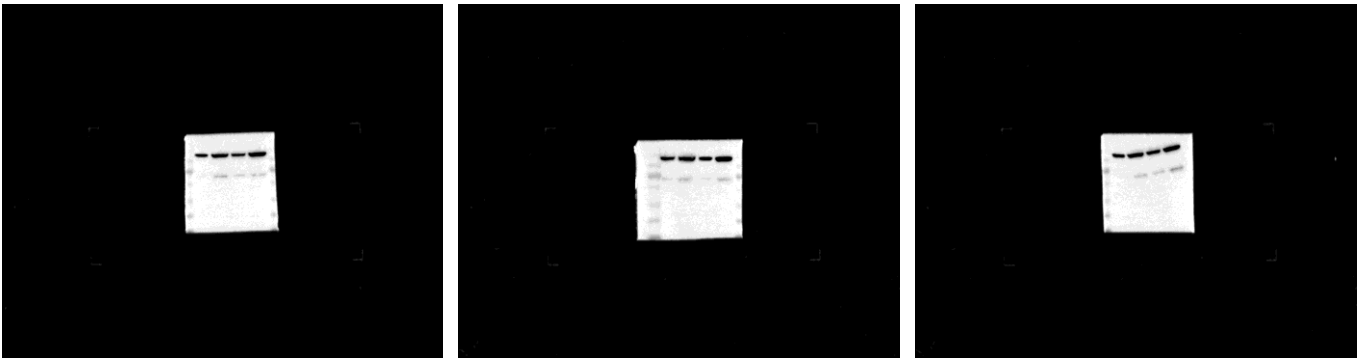

$\beta$ -actin

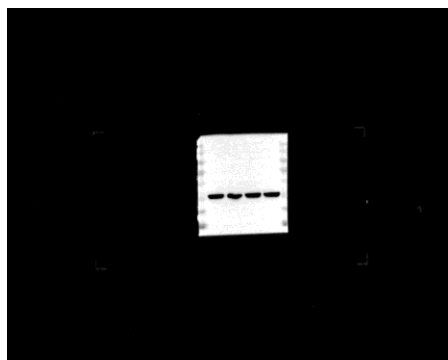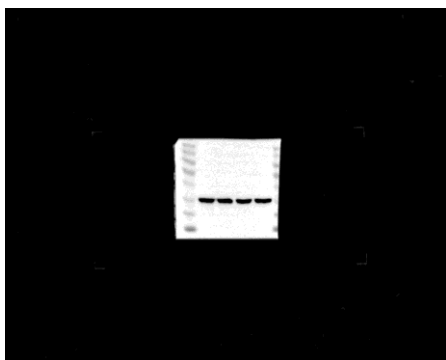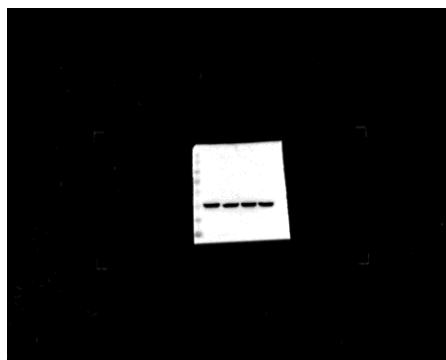

Supplement: S1 Raw Images — (PDF) [file pone.0323222.s008.pdf]
