# Supplementary material for: Sex differences in alcohol inhibits bone formation and promotes bone resorption in young male and female rats by altering intestinal flora, metabolites, and bone microenvironment
Source: PLoS One. 2025 May 8;20(5):e0323222. doi: 10.1371/journal.pone.0323222 (PMC12061194; doi:10.1371/journal.pone.0323222)
Supplement: S1 Table — (DOCX) [file pone.0323222.s003.docx]

**S1 Table.** The detection limits, and CV of intra- and inter-assays of used ELISA kits.

| Index | Detection limits | CV | |
| --- | --- | --- | --- |
|  |  | Intra-assays (%) | Inter-assays (%) |
| ADH | 0.5 μmol/L | 5.89 | 4.65 |
| OCN | 0.375 ng/mL | 2.89 | 2.84 |
| ALDH | 0.5 μmol/L | 2.37 | 1.96 |
| ALP | 6.25 ng/mL | 1.62 | 1.99 |
| TRACP-5b | 6.25 ng/mL | 4.14 | 3.28 |
| CT | 25 pg/mL | 0.93 | 3.19 |
| OPG | 125 pg/mL | 2.82 | 2.22 |
| IGF-1 | 0.75 ng/mL | 4.08 | 4.16 |

CV (%) = SD / mean × 100; intra-assays: CV < 10%; inter-assays: CV < 13%
