# Supplementary material for: Sex differences in alcohol inhibits bone formation and promotes bone resorption in young male and female rats by altering intestinal flora, metabolites, and bone microenvironment
Source: PLoS One. 2025 May 8;20(5):e0323222. doi: 10.1371/journal.pone.0323222 (PMC12061194; doi:10.1371/journal.pone.0323222)
Supplement: S2 Table — (DOCX) [file pone.0323222.s004.docx]

**S2 Table** Two-way ANOVA analysis of Figure 1.

| **Index** | **Main effect** | | | | | | **Interaction effect (sex-by-alcohol)** | | | **Multiple pairwise comparison** | | | |
| --- | --- | --- | --- | --- | --- | --- | --- | --- | --- | --- | --- | --- | --- |
|  | Sex | | | Alcohol | | |  |  |  | MN *vs.* MA | FN *vs.* FA | MN *vs.* FN | MA *vs.* FA |
|  | F | Sig. | η^2^ | F | Sig. | η^2^ | F | Sig. | η^2^ | Sig.^b^ | Sig.^b^ | Sig.^b^ | Sig.^b^ |
| Osteoclasts | 1.444 | 0.264^ns^ | 0.153 | 24.009 | 0.001^**^ | 0.750 | 1.923 | 0.203^ns^ | 0.194 | 0.038^*^ | 0.002^**^ | 0.899^ns^ | 0.105^ns^ |
| ADH | 8.026 | 0.022^*^ | 0.501 | 80.800 | 0.000^***^ | 0.910 | 9.474 | 0.015^*^ | 0.542 | 0.027^*^ | 0.000^***^ | 0.867^ns^ | 0.003^**^ |
| ALDH | 8.684 | 0.019^*^ | 0.520 | 163.061 | 0.000^***^ | 0.953 | 2.411 | 0.159^ns^ | 0.232 | 0.000^***^ | 0.000^***^ | 0.353^ns^ | 0.013^*^ |

The partial-eta-squared (η^2^) indicates the effect size, the larger the value, the larger the effect size; ^b^Bonferroni post-hoc for multiple pairwise comparisons. ^*^*p* < 0.05, ^**^*p* < 0.01, ^***^*p* < 0.001, ^ns^*p* > 0.05.
