# Supplementary material for: Sex differences in alcohol inhibits bone formation and promotes bone resorption in young male and female rats by altering intestinal flora, metabolites, and bone microenvironment
Source: PLoS One. 2025 May 8;20(5):e0323222. doi: 10.1371/journal.pone.0323222 (PMC12061194; doi:10.1371/journal.pone.0323222)
Supplement: S3 Table — (DOCX) [file pone.0323222.s005.docx]

**S3 Table** Two-way ANOVA analysis of Figure 2.

| **Index** | **Main effect** | | | | | | **Interaction effect (sex-by-alcohol)** | | | **Multiple pairwise comparison** | | | |
| --- | --- | --- | --- | --- | --- | --- | --- | --- | --- | --- | --- | --- | --- |
|  | Sex | | | Alcohol | | |  |  |  | MN *vs.* MA | FN *vs.* FA | MN *vs.* FN | MA *vs.* FA |
|  | F | Sig. | η^2^ | F | Sig. | η^2^ | F | Sig. | η^2^ | Sig.^b^ | Sig.^b^ | Sig.^b^ | Sig.^b^ |
| BALP | 36.015 | 0.000^***^ | 0.818 | 363.482 | 0.000^***^ | 0.978 | 2.282 | 0.169^ns^ | 0.222 | 0.000^***^ | 0.000^***^ | 0.013^*^ | 0.001^**^ |
| TRAP-5b | 19.432 | 0.002^**^ | 0.708 | 256.629 | 0.000^***^ | 0.970 | 14.461 | 0.005^**^ | 0.644 | 0.000^***^ | 0.000^***^ | 0.680^ns^ | 0.000^***^ |
| OCN | 16.074 | 0.004^**^ | 0.668 | 65.225 | 0.000^***^ | 0.891 | 19.787 | 0.002^**^ | 0.712 | 0.041^*^ | 0.000^***^ | 0.764^ns^ | 0.000^***^ |
| CT | 14.512 | 0.005^**^ | 0.645 | 144.351 | 0.000^***^ | 0.947 | 19.701 | 0.002^**^ | 0.711 | 0.001^**^ | 0.000^***^ | 0.668^ns^ | 0.000^***^ |
| OPG | 6.139 | 0.038^*^ | 0.434 | 539.025 | 0.000^***^ | 0.985 | 5.502 | 0.047^*^ | 0.407 | 0.000^***^ | 0.000^***^ | 0.009^**^ | 0.928^ns^ |
| IGF-1 | 4.921 | 0.057^ns^ | 0.381 | 90.748 | 0.000^***^ | 0.919 | 2.370 | 0.162^ns^ | 0.229 | 0.000^***^ | 0.000^***^ | 0.644^ns^ | 0.029^*^ |
| Ca | 3.841 | 0.086^ns^ | 0.324 | 86.056 | 0.000^***^ | 0.915 | 1.904 | 0.205^ns^ | 0.192 | 0.001^**^ | 0.000^***^ | 0.692^ns^ | 0.046^*^ |
| P | 2.413 | 0.159^ns^ | 0.232 | 54.113 | 0.000^***^ | 0.871 | 1.178 | 0.309^ns^ | 0.128 | 0.002^**^ | 0.000^***^ | 0.749^ns^ | 0.099^ns^ |

The partial-eta-squared (η^2^) indicates the effect size, the larger the value, the larger the effect size; ^b^Bonferroni post-hoc for multiple pairwise comparisons. ^*^*p* < 0.05, ^**^*p* < 0.01, ^***^*p* < 0.001, ^ns^*p* > 0.05.
