# Supplementary material for: Sex differences in alcohol inhibits bone formation and promotes bone resorption in young male and female rats by altering intestinal flora, metabolites, and bone microenvironment
Source: PLoS One. 2025 May 8;20(5):e0323222. doi: 10.1371/journal.pone.0323222 (PMC12061194; doi:10.1371/journal.pone.0323222)
Supplement: S4 Table — (DOCX) [file pone.0323222.s006.docx]

**S4 Table** Two-way ANOVA analysis of Figure 3.

| **Index** | **Main effect** | | | | | | **Interaction effect (sex-by-alcohol)** | | | **Multiple pairwise comparison** | | | |
| --- | --- | --- | --- | --- | --- | --- | --- | --- | --- | --- | --- | --- | --- |
|  | Sex | | | Alcohol | | |  |  |  | MN *vs.* MA | FN *vs.* FA | MN *vs.* FN | MA *vs.* FA |
|  | F | Sig. | η^2^ | F | Sig. | η^2^ | F | Sig. | η^2^ | Sig.^b^ | Sig.^b^ | Sig.^b^ | Sig.^b^ |
| CD80+ | 27.900 | 0.000^***^ | 0.582 | 3.826 | 0.065^ns^ | 0.161 | 12.037 | 0.002^**^ | 0.376 | 0.001^**^ | 0.297^ns^ | 0.000^***^ | 0.215^ns^ |
| CD206+ | 0.558 | 0.464^ns^ | 0.027 | 794.105 | 0.000^***^ | 0.975 | 10.450 | 0.004^**^ | 0.343 | 0.000^***^ | 0.000^***^ | 0.011^*^ | 0.094^ns^ |
| M1/M2 | 29.718 | 0.000^***^ | 0.598 | 363.461 | 0.000^***^ | 0.948 | 0.798 | 0.382^ns^ | 0.038 | 0.000^***^ | 0.000^***^ | 0.004^**^ | 0.000^***^ |
| iNOS | 4.558 | 0.065^ns^ | 0.363 | 44.342 | 0.000^***^ | 0.847 | 2.141 | 0.182^ns^ | 0.211 | 0.006^**^ | 0.000^***^ | 0.647^ns^ | 0.034^***^ |
| COX2 | 3.337 | 0.105^ns^ | 0.294 | 35.709 | 0.000^***^ | 0.817 | 4.491 | 0.067^ns^ | 0.360 | 0.026^*^ | 0.000^***^ | 0.841^ns^ | 0.024^*^ |
| CD163 | 7.949 | 0.023^*^ | 0.498 | 50.783 | 0.000^***^ | 0.864 | 2.856 | 0.129^ns^ | 0.263 | 0.005^**^ | 0.000^***^ | 0.448^ns^ | 0.013^*^ |
| Arg-1 | 0.693 | 0.429^ns^ | 0.080 | 20.394 | 0.002^**^ | 0.718 | 0.584 | 0.467^ns^ | 0.068 | 0.029^*^ | 0.006^**^ | 0.963^ns^ | 0.291^ns^ |

The partial-eta-squared (η^2^) indicates the effect size, the larger the value, the larger the effect size; ^b^Bonferroni post-hoc for multiple pairwise comparisons. ^*^*p* < 0.05, ^**^*p* < 0.01, ^***^*p* < 0.001, ^ns^*p* > 0.05.
